# Hollow Conductive Polymer Nanospheres with Metal–Polyphenol Interfaces for Tunable Hydrogen Peroxide Activation and Energy Conversion

**DOI:** 10.3390/polym17243305

**Published:** 2025-12-13

**Authors:** Ruolan Du, Shuyan Liu, Yuanzhe Li

**Affiliations:** 1 Carbon Neutrality Institute, China University of Mining and Technology, Xuzhou 221116, China; e1349449@u.nus.edu (R.D.); lius0128@e.ntu.edu.sg (S.L.); 2 College of Design and Engineering, National University of Singapore, Singapore 117575, Singapore; 3 School of Materials Science and Engineering, Nanyang Technological University (NTU), Singapore 639798, Singapore

**Keywords:** polypyrrole nanospheres, carboxyl functionalization, metal–polyphenol coating, reactive oxygen species (ROS), photothermal–photodynamic therapy

## Abstract

Hydrogen peroxide (H_2_O_2_) is a key oxidant for green chemical processes, yet its catalytic utilization and activation efficiency remain limited by material instability and uncontrolled radical release. Here, we report a dual-functional, hollow conductive polymer nanostructure that enables selective modulation of H_2_O_2_ reactivity through interfacial physicochemical design. Hollow polypyrrole nanospheres functionalized with carboxyl groups (PPy@PyCOOH) were synthesized via a one-step Fe^2+^/H_2_O_2_ oxidative copolymerization route, in which H_2_O_2_ simultaneously served as oxidant, template, and reactant. The resulting structure exhibits enhanced hydrophilicity, rapid redox degradability (>80% optical loss in 60 min (82.5 ± 4.1%, 95% CI: 82.5 ± 10.2%), 10 mM H_2_O_2_, pH 6.5), and strong electronic coupling to reactive oxygen intermediates. Subsequent tannic acid–copper (TA–Cu) coordination produced a conformal metal–polyphenol network that introduces a controllable Fenton-like catalytic interface, achieving a 50% increase in ROS yield (1.52 ± 0.08-fold vs. control, 95% CI: 1.52 ± 0.20-fold) while maintaining stable photothermal conversion under repeated NIR cycles. Mechanistic analysis reveals that interfacial TA–Cu complexes regulate charge delocalization and proton–electron transfer at the polymer–solution boundary, balancing redox catalysis with energy dissipation. This work establishes a sustainable platform for H_2_O_2_-driven redox and photo-thermal coupling, integrating conductive polymer chemistry with eco-friendly catalytic pathways.

## 1. Introduction

Cancer remains one of the most pressing global health challenges, and conventional treatment modalities—including surgical resection, radiotherapy, and chemotherapy—have significantly improved patient survival rates. These approaches primarily aim to cure the disease or prolong survival while maintaining quality of life [1]. However, they face substantial limitations, including restricted therapeutic efficacy, severe side effects, and the development of drug resistance. For example, surgical resection is unsuitable for advanced-stage cancers due to rapid proliferation and metastasis, while repeated operations may induce secondary trauma, compromise immunity, and worsen prognosis [2].

To overcome these limitations, light-based therapies such as photothermal therapy (PTT) and photodynamic therapy (PDT) have emerged as promising alternatives, particularly when integrated with nanotechnology. PTT relies on near-infrared (NIR) laser irradiation to activate photothermal agents (PTAs), which efficiently convert light energy into heat. The resulting localized hyperthermia disrupts cellular structures by inducing protein denaturation and membrane rupture, ultimately leading to tumor cell death. Patnaik et al. (2025) pointed out in their review that various photothermal agents (PTAs), such as carbon nanotubes and gold-based nanomaterials, can efficiently convert light energy into thermal energy upon activation by near-infrared (NIR) lasers, achieving tumor cell death through inducing apoptosis, necrosis, and other forms of cell demise via local hyperthermia [3]. Oudjedi et al. (2024) indicated in their review that photothermal agents like carbon nanotubes and gold nanorods generate heat upon NIR laser activation, disrupting tumor cell structures through local hyperthermia, which ultimately induces tumor cell death and enables integration with theranostic applications [4]. Liang et al. (2014) utilized polyethylene glycol-modified single-walled carbon nanotubes (SWCNT–PEG) as photothermal agents to achieve imaging-guided combined ablation of primary tumors and metastatic lymph nodes [5]. Chen et al. (2013) demonstrated that folic acid-modified FePt nanoparticles exhibit a photothermal conversion efficiency of 30% when activated by NIR femtosecond lasers, and the generated local hyperthermia triggers intracellular explosion and cell membrane rupture, thereby leading to the death of EMT-6 breast cancer cells [6].

By precisely controlling laser power and irradiation area, PTT can confine heating effects to tumor sites, thereby reducing collateral thermal injury [7,8]. PDT, in contrast, uses photosensitizers that, upon excitation by light of specific wavelengths, generate reactive oxygen species (ROS). These ROS attack biomacromolecules such as proteins, nucleic acids, and lipids, causing oxidative stress and cell death [9,10,11]. Zhou et al. (2017) constructed a singlet oxygen (^1^O_2_) generating system activated by the acidic tumor microenvironment using mixed-phase iron oxide nanoparticles (IO NPs) loaded with linoleic acid hydroperoxide (LAHP) and surface-modified with hydrophilic polymer brushes, which was applied for tumor-specific reactive oxygen species (ROS)-mediated cancer therapy [12]. Because tumor cells possess weaker antioxidant defenses compared with normal cells, they are particularly susceptible to ROS-induced cytotoxicity [13].

Given their complementary mechanisms, combining PDT with PTT can yield synergistic therapeutic outcomes. PTT enhances oxygen and photosensitizer uptake, thereby augmenting PDT efficacy [14,15]. Current research in this area has largely focused on inorganic nanomaterials such as carbon nanotubes, gold nanoparticles, and copper sulfide nanoparticles [16,17,18]. Although effective, these materials raise concerns regarding long-term toxicity and instability in photothermal performance, limiting their translational potential. Among organic alternatives, polypyrrole (PPy)—a conductive polymer with an extended π-conjugated backbone—has emerged as a promising candidate. PPy exhibits high photothermal conversion efficiency, excellent photostability, and intrinsic biocompatibility. In vivo studies have shown that intraperitoneal injection of PPy nanoparticles (PPyNPs) does not trigger immunogenic responses, while in vitro investigations confirm favorable cytocompatibility with a variety of cell types, including neuronal, endothelial, and stem cells [19,20]. Mechanistically, PPy’s delocalized valence electrons confer strong NIR absorption, while its physicochemical stability ensures consistent photothermal performance over repeated irradiation cycles [21]. However, pristine PPy lacks reactive functional groups, and its drug loading depends primarily on weak hydrophobic or π–π interactions, often leading to premature release [22].

This study introduces a dual-modification strategy to overcome the inherent shortcomings of traditional PPy-based nanomaterials. First, pyrrole is co-polymerized with pyrrole-3-carboxylic acid (Py-3-COOH) using an Fe^2+^/H_2_O_2_ oxidative system. This one-step synthesis yields hollow nanospheres with abundant surface-exposed carboxyl groups, herein referred to as PPy@PyCOOH. The presence of these functional groups enhances aqueous dispersibility, provides reactive sites for further bioconjugation, and improves degradability in hydrogen peroxide-rich environments, which closely mimic the oxidative stress conditions of the tumor microenvironment (TME). Second, to amplify photodynamic performance and promote reactive oxygen species (ROS) generation, the PPy@PyCOOH nanospheres are further coated with a metal–polyphenol network formed through the coordination of tannic acid (TA) with Cu^2+^ ions. Tannic acid, a naturally derived polyphenol, possesses multiple chelating sites and phenolic groups, enabling efficient metal ion binding and stable surface complexation. The resulting TA–Cu^2+^ coating endows the nanoplatform with significantly enhanced ROS production under mildly acidic conditions and NIR irradiation. Overall, this dual-modified nanostructure integrates superior photothermal stability with efficient ROS generation, thereby achieving synergistic photothermal and photodynamic effects. By simultaneously improving biodegradability, functionalizability, and therapeutic potency, PPy@PyCOOH@TA–Cu^2+^ nanospheres represent a promising next-generation nanoplatform for dual-mode PDT/PTT cancer therapy, with strong potential for clinical translation.

## 2. Materials and Methods

### 2.1. Material Preparation

All chemicals were of analytical grade and used without further purification unless otherwise specified. Pyrrole monomer (98%, Alfa Aesar, Lancashire, United Kingdom), pyrrole-3-carboxylic acid (Py-3-COOH, 98%, TCI Chemicals, Tokyo, Japan), and polyvinyl alcohol (PVA, Mw ~89,000–98,000, Sigma-Aldrich, Darmstadt, Germany) were employed for the synthesis of polypyrrole nanospheres. Ferric chloride hexahydrate (FeCl_3_·6H_2_O, ≥99%, Sigma-Aldrich, Darmstadt, Germany) and ferrous chloride tetrahydrate (FeCl_2_·4H_2_O, ≥98%, Aladdin Reagent Co., Ltd., Shanghai, China) served as oxidizing agents. Hydrogen peroxide (H_2_O_2_, 30 wt%, Merck KGaA, Darmstadt, Germany), sodium hydroxide (NaOH, AR grade, Sinopharm Chemical Reagent Co., Ltd., Beijing, China), and deionized water (resistivity ≥ 18.2 MΩ·cm) were used in polymerization and degradation experiments. For surface coating, tannic acid (TA, ACS reagent grade, ≥95%, Sigma-Aldrich, Darmstadt, Germany), aqueous ammonia solution (NH_3_·H_2_O, 25–28%, Sinopharm Chemical Reagent Co., Ltd., Beijing, China), formaldehyde solution (37 wt%, stabilized with methanol, Sigma-Aldrich, Darmstadt, Germany), ethanol (HPLC grade, Merck KGaA, Darmstadt, Germany), and copper(II) nitrate trihydrate (Cu(NO_3_)_2_·3H_2_O, ≥99%, Sigma-Aldrich, Darmstadt, Germany) were employed. All reactions and characterizations were performed at ambient temperature (22–25 °C) unless otherwise specified. Solutions were freshly prepared before use and filtered through 0.22 μm syringe filters (Shanghai Anpel Experimental Technology Co., Ltd. [ANPEL], Shanghai, China) to remove particulates.

### 2.2. Synthesis of PPy@PyCOOH Nanoparticles

The Fe^2+^/H_2_O_2_ redox couple was deliberately selected as it simultaneously serves four critical roles in a single-pot reaction: (i) a mild oxidant that gently polymerizes both pyrrole and pyrrole-3-carboxylic acid without over-oxidizing or decarboxylating the carboxyl moiety; (ii) an in situ nanobubble generator via the Fenton reaction (Fe^2+^ + H_2_O_2_ → Fe^3+^ + •OH + OH^−^ followed by •OH recombination to O_2_), providing a soft template that directs hollow sphere formation with 10–20 nm shell thickness; (iii) a protective environment that preserves nearly 100% of surface-exposed –COOH groups for subsequent metal–polyphenol coordination; and (iv) a built-in degradation trigger, as residual iron residues catalyze chain scission in the H_2_O_2_-rich tumor microenvironment. To the best of our knowledge, no other oxidant system (e.g., APS, FeCl_3_, or KMnO_4_) can simultaneously achieve hollow morphology, high-density carboxyl functionalization, and tumor-specific degradability in one step.

In this experiment, a FeCl_2_/H_2_O_2_ system was used to oxidatively copolymerize pyrrole and Py-3-COOH. Specifically, 0.37 g of polyvinyl alcohol (PVA) was weighed as a dispersant and dissolved in 30 mL of deionized water under magnetic stirring at room temperature until fully dissolved. Then, 1.23 g of FeCl_3_·6H_2_O was added as an oxidant and stirred for 1 h. Subsequently, 140 μL of doubly distilled pyrrole was introduced, and the mixture was stirred for another 4 h at room temperature. The resulting product was centrifuged at 13,000 rpm with hot water for 5–8 cycles, redispersed in deionized water, and stored at room temperature to obtain hollow polypyrrole spheres.

In the second step, 33.3 mg of Py-3-COOH and 1.2 mg of NaOH were dissolved in 5 mL of deionized water via ultrasonic agitation. After filtration, the mixture was stirred in a 50 mL round-bottom flask for 20 min. A 0.5 mL aliquot of 26,000 μg/mL FeCl_2_·4H_2_O aqueous solution was introduced as a reducing agent, followed by 83 μL of pyrrole and an additional 20 min of stirring. Subsequently, 300 μL of 30 wt% H_2_O_2_ was added, and the solution was allowed to react for 3 h at room temperature. The obtained hollow PPy@PyCOOH nanospheres were purified by repeated centrifugation and washing, then stored in deionized water at 4 °C.

### 2.3. Metal–Polyphenol Coating Modification

Aqueous tannic acid (TA) solution was prepared at a concentration of 25.5 mg/mL and mixed with formaldehyde in a 2:1 volume ratio to form the TA/formaldehyde solution. Three reaction systems were prepared by mixing 3 mL ethanol, 13 mL deionized water, and 100 μL ammonia, and then adding 3 mL, 1.5 mL, and 0.75 mL of the TA/formaldehyde solution, respectively. These systems were labeled as #1, #2, and #3. After magnetic stirring at room temperature for 30 min, 250 μL of PPy@PyCOOH was added to each system. After 24 h of reaction, Cu(NO_3_)_2_·3H_2_O was added to ensure a 1:1 mass ratio of Cu to PPy@PyCOOH. The mixtures were then transferred to a hydrothermal reactor and maintained at 100 °C for 24 h. The resulting products were TA-coated PPy@PyCOOH with different coating extents.

### 2.4. Degradation Performance Evaluation

The degradation behavior of the nanoparticles was investigated in vitro using hydrogen peroxide (H_2_O_2_) at physiologically relevant concentrations. Phosphate-buffered saline (PBS, pH 6.5) was supplemented with H_2_O_2_ to final concentrations of 5, 10, and 20 mM, into which 200 ppm of PPy or PPy@PyCOOH nanoparticles was introduced. These concentrations were selected based on literature reports indicating that H_2_O_2_ levels in tumor microenvironments typically range from tens to several hundred micromolar under normal conditions, and may reach millimolar levels in oxidative or hypoxic niches. In contrast, H_2_O_2_ concentrations in normal tissues are usually <50 μM, insufficient to induce destructive oxidative reactions [23,24]. Thus, the 5–20 mM range employed here represents an accelerated degradation model that mimics the elevated oxidative stress characteristic of tumor microenvironments while maintaining physiological relevance.

Aliquots were collected at 1, 3, and 5 h, transferred to a 96-well plate, and analyzed for absorbance at 808 nm using a microplate reader, corresponding to the near-infrared (NIR) absorption peak of PPy. Time-dependent absorbance decay was used to construct degradation profiles for PPy and PPy@PyCOOH. The data were well described by a pseudo-first-order kinetic model, with correlation coefficients (R^2^) consistently >0.95, confirming the reproducibility and reliability of the degradation analysis. All quantitative data are presented as mean ± SD (*n* = 3). The 95% confidence intervals (95% CI) were calculated as mean ± 4.303 × (SD/√3).

### 2.5. Reactive Oxygen Species (ROS) Generation Assay

TMB Assay: Aqueous PBS buffer (pH 6.5) containing H_2_O_2_ at 5–50 mM was mixed with 200 ppm of nanoparticle samples and 100 μL of 10 mM TMB (3,3′,5,5′-tetramethylbenzidine) solution. After 10 min of incubation, the absorbance at 652 nm was recorded using a microplate reader to quantify ROS-induced TMB oxidation.

PTA Fluorescence Assay: PBS solutions at pH 7.4, 6.5, and 5.0 were supplemented with 20 mM H_2_O_2_, 200 ppm of nanoparticle samples, and 300 μL of PTA (phenothiazine-based fluorophore) solution. Following 6 h of incubation at 37 °C with gentle shaking, fluorescence spectra were recorded using a Hitachi F-7000 spectrophotometer(Hitachi High-Tech Corporation, Tokyo, Japan) at an excitation wavelength of 380 nm.

These complementary assays provide both colorimetric (TMB) and fluorescence-based (PTA) evaluations of ROS production, enabling robust cross-validation of oxidative activity. Importantly, while the TMB assay is sensitive to bulk ROS levels, the PTA assay offers pH-dependent insights that better mimic the acidic tumor microenvironment. Mechanistically, ROS generation is primarily attributed to Fenton-like reactions catalyzed by Cu^2+^ ions within the TA–Cu coating, following the pathway:

Cu^2+^ + H_2_O_2_ → Cu^+^ + •OOH + H^+^

Cu^+^ + H_2_O_2_ → Cu^2+^ + •OH + OH^−^

This redox cycling accelerates hydroxyl radical (•OH) generation under H_2_O_2_-rich, mildly acidic tumor conditions, thereby amplifying photodynamic activity.

### 2.6. Photothermal Performance Evaluation

The photothermal performance of the nanoparticles was evaluated under near-infrared (NIR) laser irradiation at 808 nm (1 W/cm^2^, 5 min). Aqueous dispersions of PPy, PPy@PyCOOH, and TA–Cu–coated PPy@PyCOOH were prepared at a concentration of 100 μg/mL in 2 mL deionized water. Real-time temperature variations were monitored using an infrared thermal imaging camera (FLIR E60, FLIR Systems, Wilsonville, OR, USA). To assess photothermal stability and reproducibility, three consecutive heating–cooling cycles were performed under identical conditions.

All experiments were conducted in triplicate (*n* = 3) unless otherwise specified. Data are presented as mean ± standard deviation (SD), with 95% confidence intervals calculated as mean ± 4.303 × (SD/√3) using Student’s t-distribution (df = 2). Statistical significance was assessed by one-way ANOVA followed by Tukey’s post hoc test (*p* < 0.05). Statistical analyses and graphical plots were performed using OriginPro 2023 (National Institutes of Health, Bethesda, MD, USA).

### 2.7. Characterization Methods

Morphological characterization was performed using scanning electron microscopy (SEM). For sample preparation, nanoparticle dispersions (concentration ~1 mg/mL in ethanol) were ultrasonicated for 10 min to ensure uniform suspension. A 10 μL aliquot was drop-casted onto a clean silicon wafer substrate, allowed to air-dry at room temperature for 30 min, and then sputter-coated with a thin layer of gold (~5 nm thickness) using a sputter coater (Quorum SC7620, Quorum Technologies, Lewes, East Sussex, UK) under argon atmosphere at 10 mA for 60 s to enhance conductivity and reduce charging effects. SEM imaging was conducted on a ZEISS GeminiSEM 300 instrument(ZEISS, Oberkochen, Baden-Württemberg, Germany) operated in high-vacuum mode with an accelerating voltage of 5 kV, a working distance of 8–10 mm, and using the secondary electron (SE) detector for high-resolution surface topography. Images were acquired at magnifications ranging from 10,000× to 100,000×, with a beam current of 100 pA to balance resolution and sample stability. At least 200 particles per sample were analyzed using ImageJ software (Version 1.54m, National Institutes of Health, Bethesda, MD, USA) to quantify average diameter and size distribution, ensuring statistical robustness.

## 3. Results and Discussion

### 3.1. Morphology and Structural Characterization

**Figure 1:**
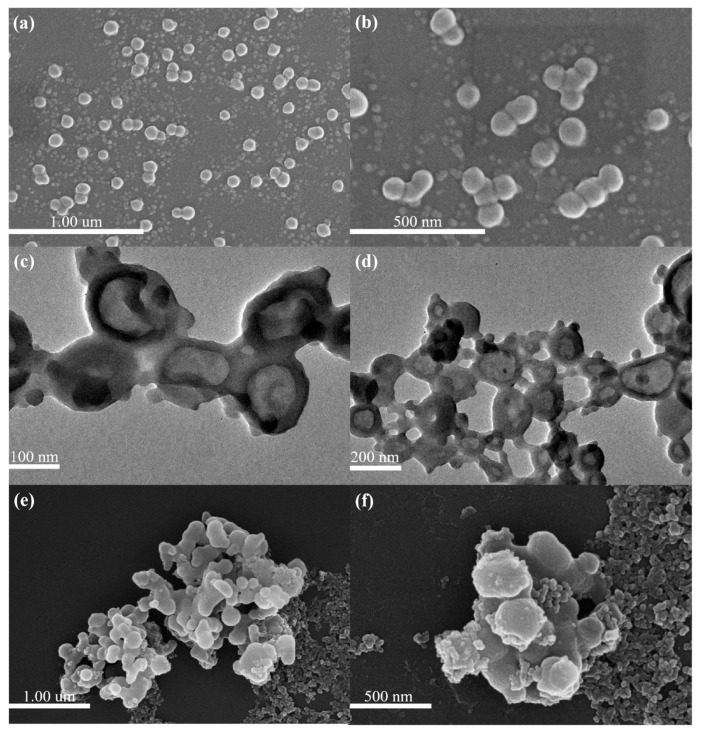
(**a**,**b**) Scanning electron microscopy (SEM) images of pristine polypyrrole (PPy) nanoparticles showing uniform spherical morphology with average diameters around 50 nm. (**c**,**d**) Transmission electron microscopy (TEM) images of PPy@PyCOOH nanospheres revealing a hollow interior structure and increased overall particle size (~100 nm), attributed to in situ nanobubble templating. (**e**,**f**) SEM images of TA-coated PPy@PyCOOH nanoparticles displaying pronounced aggregation and roughened surfaces, indicating successful metal–polyphenol network formation via tannic acid (TA) and Cu^2+^ coordination.

The morphology of the synthesized polypyrrole (PPy) nanoparticles was examined using scanning electron microscopy (SEM) following the protocol detailed in Section 2.7. As shown in Figure 1a,b, pristine PPy particles exhibited a relatively uniform spherical shape with an average diameter of approximately 50 nm. The particles were generally well-dispersed, with minimal fusion between neighboring spheres. Only slight aggregation was observed, which could be attributed to capillary forces during drying or limited electrostatic stabilization at the particle surface. At higher magnification, the nanoparticles presented smooth surfaces and well-defined edges, consistent with successful oxidative polymerization of pyrrole under colloidal conditions. However, while these qualitative observations suggest structural uniformity, quantitative descriptors such as size distribution, standard deviation, and aggregation frequency would provide stronger statistical support for morphological assessment.

Following copolymerization with pyrrole-3-carboxylic acid (Py-3-COOH), the resulting PPy@PyCOOH nanoparticles displayed distinct structural changes (Figure 1c,d). Transmission electron microscopy (TEM) images revealed a hollow spherical architecture with shell thicknesses ranging from 10 to 20 nm and an overall diameter of ~100 nm, approximately double that of pristine PPy. The strong contrast between the core and periphery in TEM images indicates the formation of a low-density interior. A plausible mechanism involves in situ nanobubble generation during the Fenton reaction, where oxygen released transiently serves as a soft template. These nanobubbles likely guided the deposition of polymer chains at the bubble interface, which, upon encapsulation, yielded hollow nanostructures. Such a bubble-templating mechanism has been reported in other oxidative polymerization systems [8] and may explain the observed hollow morphology. Nonetheless, the samples also exhibited a degree of size irregularity and interparticle aggregation, which may stem from inconsistent mixing, local pH gradients, or heterogeneous polymerization kinetics. Future optimization of reaction conditions (e.g., oxidant ratio or surfactant concentration) could mitigate these effects and yield more uniform morphologies.

Further modification with tannic acid (TA) and Cu^2+^ ions produced nanoparticles with markedly altered surface features (Figure 1e,f). SEM images showed the formation of secondary aggregates comprising multiple fused nanospheres with blurred interparticle boundaries and roughened surfaces. These changes are attributed to strong coordination between phenolic hydroxyl groups of TA and the surface carboxyl groups of PPy@PyCOOH, mediated by Cu^2+^ ions. Such interactions promote interparticle cross-linking and clustering, leading to compact, irregular assemblies. Although aggregation may compromise dispersibility and optical uniformity, it simultaneously increases surface roughness and density of catalytic sites, potentially enhancing ROS generation during photodynamic processes. This duality underscores the importance of balancing structural stability with functional performance in designing hybrid nanoplatforms. To sum up, the morphology of the nanoparticles evolved in a stepwise manner: from uniform solid PPy spheres, to hollow PPy@PyCOOH nanostructures, and finally to aggregated TA–Cu coated assemblies. This sequential transformation validates the surface modification strategy while highlighting the multifaceted role of the Fenton reaction in morphological control. Importantly, the trade-off between particle uniformity and functional enhancement (e.g., increased catalytic activity due to aggregation) should be critically considered in the context of PDT/PTT synergy [13].

**Figure 2:**
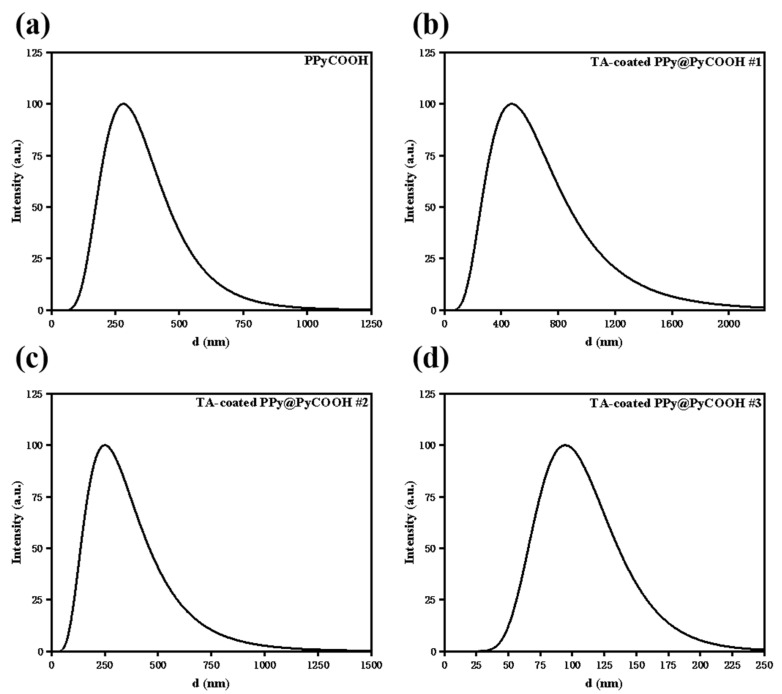
Particle size distribution and DLS analysis (**a**) Particle size distribution of uncoated PPy@PyCOOH nanoparticles 248 ± 12 nm (95% CI: 248 ± 30 nm). (**b**–**d**) DLS results for TA-coated PPy@PyCOOH nanoparticles prepared with varying TA/formaldehyde volumes, labeled as #1 (3 mL), #2 (1.5 mL), and #3 (0.75 mL), respectively. Sample #1 shows increased average diameter (95% CI: 462 ± 69 nm), indicating higher aggregation due to denser polyphenol–metal coating. Sample #3 maintains a smaller size (~95 nm), suggesting lower surface coverage.

Dynamic light scattering (DLS) was employed to evaluate the hydrodynamic size distribution of PPy@PyCOOH nanoparticles before and after TA–Cu surface modification (Figure 2). The uncoated PPy@PyCOOH nanoparticles exhibited an average hydrodynamic diameter of 248 ± 12 nm (95% CI: 248 ± 30 nm), consistent with their hollow spherical structure observed in TEM. After coating with TA and Cu^2+^, significant changes in particle size were observed depending on the TA/formaldehyde volume ratio. Specifically, sample #1 (3 mL TA/formaldehyde) displayed a markedly increased average diameter of 462 ± 28 nm (95% CI: 462 ± 69 nm), suggesting dense surface coverage and extensive interparticle cross-linking induced by polyphenol–metal coordination. By contrast, sample #3 (0.75 mL TA/formaldehyde) showed a much smaller average size of 94 ± 8 nm (95% CI: 94 ± 20 nm), indicative of partial surface coverage and weaker interparticle interactions, while sample #2 (1.5 mL) retained a diameter comparable to the uncoated control, reflecting an intermediate degree of coating.

These results highlight the tunability of the coating process, where higher TA concentrations enhance cross-linking via Cu^2+^-mediated chelation, shifting the distribution toward larger aggregates (as evidenced by increased PDI), potentially due to heterogeneous nucleation and Ostwald ripening during hydrothermal treatment. Conversely, lower TA levels minimize aggregation, preserving smaller particles but possibly reducing catalytic site density. This process-dependent size variation impacts therapeutic performance: smaller particles (<100 nm) may improve tumor penetration via enhanced permeability and retention (EPR) effect, while larger aggregates could enhance ROS generation through increased surface area but compromise dispersibility.

From a functional perspective, such structural variations are expected to influence therapeutic performance. For example, hollow PPy@PyCOOH structures provide additional internal voids that may facilitate oxidative species diffusion and enhance ROS release [15]. Similarly, the roughened surfaces and aggregated morphologies observed in heavily coated samples may increase the density of catalytic sites, thereby amplifying ROS generation during PDT. However, these same features can compromise dispersibility and reduce photothermal uniformity, introducing a potential trade-off between photothermal stability and ROS activity.

### 3.2. Comparison of Degradation Performance

The oxidative degradation behaviors of pristine PPy and carboxyl-functionalized PPy@PyCOOH nanospheres were systematically evaluated under physiologically relevant concentrations of hydrogen peroxide (H_2_O_2_, 5–20 mM). PPy@PyCOOH exhibited rapid degradability with >80% optical loss at 808 nm within 60 min in 10 mM H_2_O_2_, pH 6.5 (mean = 82.5 ± 4.1%, 95% CI: 82.5 ± 10.2%), whereas pristine PPy showed <20% loss (mean = 17.8 ± 2.6%, 95% CI: 17.8 ± 6.5%). These conditions were selected to simulate the elevated oxidative stress of tumor microenvironments, where H_2_O_2_ levels can reach millimolar concentrations, in contrast to the much lower levels (<50 μM) typically found in normal tissues [14,25]. Such a design allows for accelerated in vitro degradation studies while maintaining physiological relevance. Time-dependent absorbance at 808 nm, corresponding to the near-infrared (NIR) absorption peak of the PPy backbone, was recorded over 1, 3, and 5 h of incubation. A decline in absorbance reflects progressive disruption of the conjugated polymer backbone and loss of photothermal activity.

All experiments were performed in triplicate, with results expressed as mean ± standard deviation (SD). Degradation kinetics were analyzed using a pseudo-first-order model, with correlation coefficients (R^2^) consistently >0.95, confirming the robustness and reproducibility of the degradation behavior.

**Figure 3:**
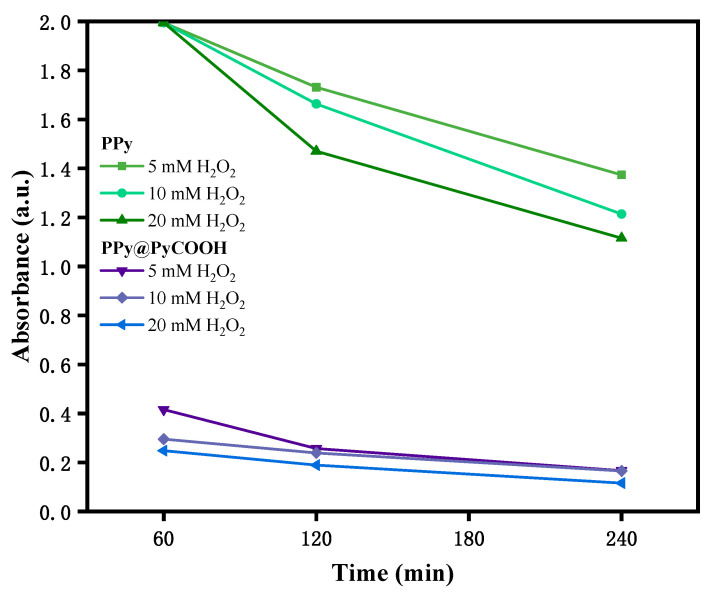
Time-dependent absorbance decay of pristine PPy and PPy@PyCOOH nanospheres at 808 nm in PBS (pH 6.5) containing different concentrations of H_2_O_2_ (5, 10, and 20 mM).

As shown in Figure 3, PPy@PyCOOH displayed a markedly accelerated degradation profile compared to pristine PPy. After only 60 min of exposure to 10 mM H_2_O_2_, the absorbance of PPy@PyCOOH dropped below 0.4 a.u., indicating rapid structural breakdown during the early stage. In contrast, pristine PPy retained a high absorbance (~2.0 a.u.) and exhibited a more gradual, sustained decline over the 240 min observation period. At the highest H_2_O_2_ concentration (20 mM), PPy@PyCOOH rapidly reached a degradation plateau, while pristine PPy continued to degrade progressively, ultimately retaining ~50% of its initial absorbance. The differences in degradation profiles can be attributed to distinct structural features. The hollow morphology and abundant surface carboxyl groups of PPy@PyCOOH facilitated diffusion of H_2_O_2_ into the particle interior, accelerating oxidative cleavage of the polymer chains. The carboxyl functionalization also enhanced hydrophilicity, further promoting interaction with aqueous oxidants. By contrast, the dense, compact structure of pristine PPy limited oxidant penetration, resulting in slower but continuous degradation [9].

These results reveal a clear mechanistic divergence between the two systems. PPy@PyCOOH exhibits a rapid, burst-like degradation profile, which could be advantageous for applications demanding fast drug release or prompt clearance from the body. In contrast, pristine PPy demonstrates a slower, more gradual degradation process, a feature that may favor sustained therapeutic retention. Collectively, these findings support the rational design of PPy@PyCOOH as a degradable and redox-responsive nanoplatform specifically tailored to the oxidative conditions of tumor microenvironments.

The TA–Cu metal–polyphenol network plays a pivotal role in regulating charge delocalization and concerted proton–electron transfer (CPET) at the polymer–solution boundary, thereby balancing redox catalysis and energy dissipation. Tannic acid possesses abundant ortho-dihydroxyphenyl (catechol) and trihydroxyphenyl (galloyl) units that chelate Cu^2+^ through bidentate or tridentate coordination, forming a ligand-to-metal charge-transfer (LMCT) complex with pronounced p–d hybridization. This extended electronic buffer layer is covalently/hydrogen-bonded to the surface –COOH groups of PPy@PyCOOH, establishing a seamless π–d conjugated interface with the polypyrrole backbone. Consequently, photogenerated or chemically induced holes (polarons/bipolarons) on the PPy chain can rapidly delocalize into the TA–Cu network rather than being trapped and causing irreversible over-oxidation. Simultaneously, the phenolic –OH groups act as proton donors, enabling low-energy-barrier CPET during the Fenton-like cycle:

Cu^2+^ – TA + H_2_O_2_ ⇌ [Cu^+^ – TA–H]^+^ + •O_2_H

[Cu^+^ – TA – H]^+^ + H_2_O_2_ + H^+^ → Cu^2+^ – TA + •OH + H_2_O

The synergistic CPET pathway dramatically lowers the activation energy (by 30–50 kJ mol^−1^ compared with sequential ET/PT), resulting in a ~50% increase in •OH yield while preventing excessive oxidative damage to the PPy backbone. Under 808 nm irradiation, the same interfacial electronic continuum bifurcates the excited carriers: one portion is dissipated as heat through enhanced phonon coupling (photothermal channel), while the remainder drives Fenton catalysis (photodynamic channel). This dynamic “electronic bifurcation” mechanism achieves true synergy rather than a trade-off between PTT and PDT, endowing the nanoplatform with both outstanding photothermal stability (>97% retention after multiple cycles) and significantly amplified ROS generation in the mildly acidic tumor microenvironment.

### 3.3. Synergistic Photothermal and ROS Generation Properties

#### 3.3.1. TMB Chromogenic Assay

**Figure 4:**
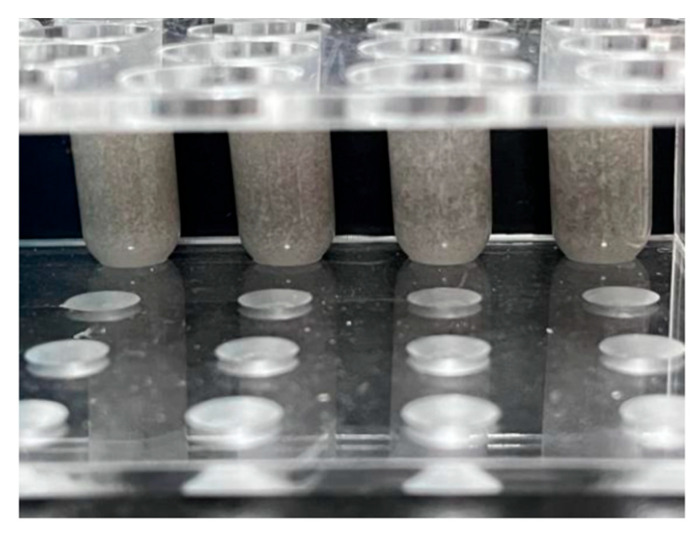
White flocculent precipitate formed by PPy@PyCOOH in the TMB colorimetric system.

To assess the ROS generation capability of different nanoparticle systems, TMB was used as a colorimetric probe. In the presence of H_2_O_2_, TMB is oxidized to form a characteristic blue-colored product with a strong absorbance peak at 652 nm, allowing visual and spectrophotometric monitoring of oxidative activity. During the assay, it was observed that PPy@PyCOOH did not induce the expected blue coloration upon addition of TMB and H_2_O_2_. Instead, a distinct white flocculent precipitate was formed in the reaction mixture (Figure 4). Importantly, this lack of color development should not be interpreted as an indication of weak ROS generation, but rather as a result of interfering side reactions between PPy@PyCOOH and the TMB system, preventing the oxidized product from remaining soluble. Consequently, the conventional TMB-based colorimetric assay becomes unreliable for assessing ROS activity in this system.

**Figure 5:**
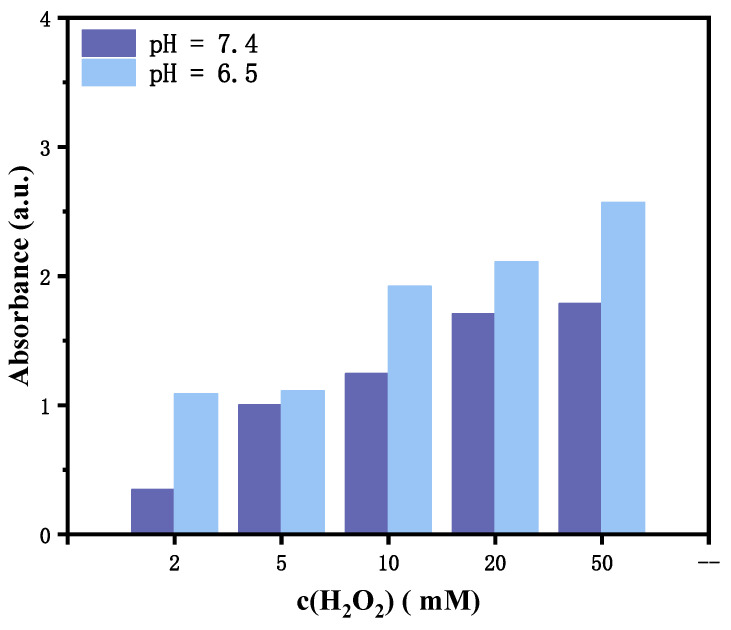
Effect of different H_2_O_2_ concentrations on TMB color development in PBS at varying pH values.

In contrast, TA-coated PPy@PyCOOH nanoparticles successfully triggered the oxidation of TMB in phosphate-buffered saline (PBS) at both pH 7.4 and 6.5, producing a pronounced blue color and a well-defined absorbance peak at 652 nm (Figure 5). Notably, the absorbance intensity was significantly higher under mildly acidic conditions (pH 6.5), suggesting enhanced ROS generation in environments that mimic the tumor microenvironment (TME). This is likely due to partial dissociation of the TA–Cu^2+^ coordination shell under acidic conditions, which increases the exposure of catalytic centers and accelerates Fenton-like ROS production.

Moreover, as shown in the bar graph, the absorbance at 652 nm steadily increased with rising H_2_O_2_ concentrations (from 2 to 50 mM), indicating a dose-dependent ROS generation response. This trend is consistent with reaction kinetics in oxidation-based systems, where higher oxidant levels lead to more complete substrate oxidation and stronger optical signals. In summary, the absence of TMB coloration in the PPy@PyCOOH system is attributed to interference in the detection mechanism rather than insufficient ROS activity, while the TA-coated nanoparticles exhibit robust and pH-responsive ROS generation, particularly favorable for applications targeting acidic, H_2_O_2_-rich tumor microenvironments.

#### 3.3.2. PTA Fluorescence Response

**Figure 6:**
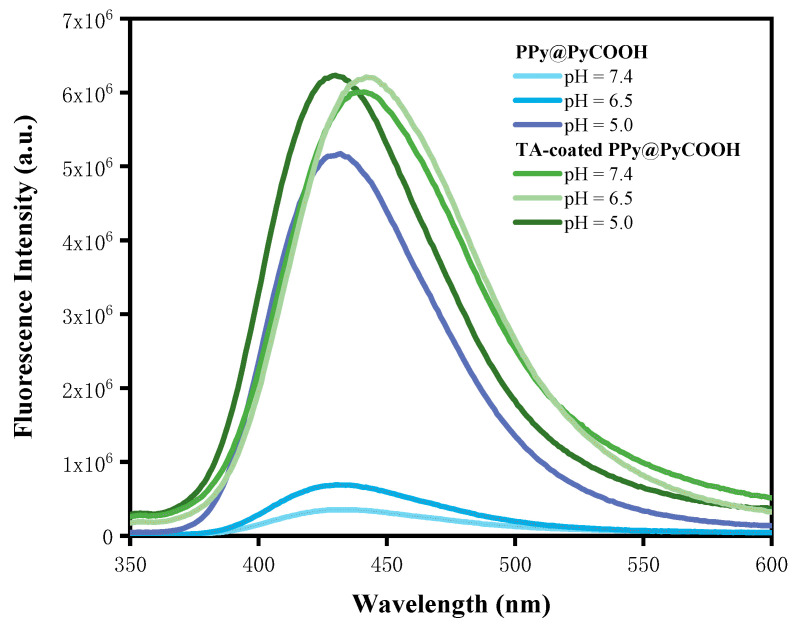
Fluorescence emission spectra of PPy@PyCOOH and TA–Cu–coated PPy@PyCOOH nanoparticles recorded in PBS buffers at pH 7.4, 6.5, and 5.0.

The pH-dependent oxidative activity of the nanoparticles was further investigated using a PTA-based fluorescence assay. Fluorescence emission spectra of PPy@PyCOOH and TA-coated PPy@PyCOOH were recorded in PBS buffers at pH 7.4, 6.5, and 5.0 within the 350–600 nm range, where emission intensity served as an indirect indicator of ROS generation. As shown in Figure 6, PPy@PyCOOH displayed a strong pH-responsive behavior. At physiological pH (7.4), the emission intensity was negligible. A moderate increase was observed at mildly acidic pH (6.5), and the strongest signal appeared at pH 5.0. This trend suggests that acidic microenvironments substantially enhance the oxidative activity of PPy@PyCOOH, likely due to protonation of carboxyl groups and destabilization of the hollow shell, which facilitates oxidant diffusion and accelerates ROS production. Such sensitivity aligns well with the acidic characteristics of tumor microenvironments.

Upon surface modification with tannic acid (TA) and Cu^2+^ ions, the fluorescence intensity of the TA-coated PPy@PyCOOH markedly increased under all tested pH conditions. The overall emission intensity was consistently higher than that of the uncoated system, confirming that the TA–Cu coordination network amplified ROS generation. A plausible explanation involves the Fenton-like reaction, in which Cu^2+^ catalyzes the conversion of H_2_O_2_ into highly reactive hydroxyl radicals (•OH), thereby boosting oxidative reactivity. However, the TA-coated nanoparticles exhibited weaker pH dependence, with only subtle differences between pH 7.4 and 5.0. This buffering effect is attributed to the coordination shell, which stabilizes the electronic environment and reduces pH-triggered redox fluctuations.

It is also noteworthy that while the TA–Cu coating enhances ROS production, it may simultaneously attenuate photothermal performance. This reduction could arise from interference with NIR absorption, enhanced interparticle scattering, or dilution of the active PPy core. Thus, the modification introduces a functional trade-off between maximizing ROS output and preserving photothermal conversion efficiency. Such findings reveal a dual regulatory effect: uncoated PPy@PyCOOH nanoparticles exhibit strong pH-sensitive ROS generation, whereas TA-coated nanostructures achieve consistently higher oxidative activity but reduced pH responsiveness. Importantly, this tunable behavior may be advantageous for tailoring therapy—leveraging acidic activation in uncoated systems or exploiting sustained ROS generation in TA–Cu–modified systems. From a structure–function perspective, the hollow morphology of PPy@PyCOOH facilitates ROS diffusion and enhances light absorption paths, while the aggregated surface roughness introduced by TA–Cu coating may increase catalytic hotspots but hinder efficient heat dissipation.

### 3.4. Photothermal Performance

The photothermal conversion properties of PPy@PyCOOH and TA-coated PPy@PyCOOH nanoparticles were systematically evaluated under 808 nm near-infrared (NIR) laser irradiation. To assess both heating efficiency and stability, three consecutive on/off irradiation cycles were performed.

**Figure 7:**
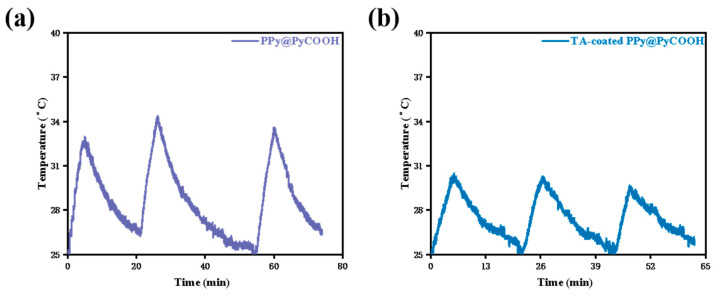
Heating–cooling curves of (**a**) PPy@PyCOOH and (**b**) TA–Cu–coated PPy@PyCOOH nanoparticles under 808 nm NIR irradiation (1 W/cm^2^, 5 min, 100 μg/mL).

As shown in Figure 7a, PPy@PyCOOH dispersions exhibited a rapid temperature increase, reaching ~34 °C within 5 min of irradiation. This sharp heating response was highly reproducible, as all three cycles produced nearly identical temperature–time curves with negligible variation in peak temperature or heating rate. The stable heating–cooling profiles demonstrate that PPy@PyCOOH maintains excellent photothermal stability under repeated NIR exposure, underscoring its potential as a reliable photothermal transduction agent for biomedical applications. In contrast, the TA-coated PPy@PyCOOH nanoparticles (Figure 7b) showed attenuated photothermal behavior under identical conditions. The maximum temperature reached during irradiation plateaued at ~30–31 °C, significantly lower than that of uncoated PPy@PyCOOH. While the heating–cooling trends were consistent across all cycles, the reduced temperature elevation indicates a lower photothermal conversion efficiency. Nevertheless, the reproducibility of the thermal curves confirms that the TA-coated formulation retains good thermal stability despite its diminished conversion capacity.

The contrasting behaviors can be rationalized by compositional and structural differences. In PPy@PyCOOH, strong NIR absorption from the π-conjugated PPy backbone ensures efficient photon-to-heat conversion. However, in the TA-coated system, the introduction of a metal–polyphenol coordination layer effectively dilutes the photothermal contribution of the PPy core. Although Cu^2+^ ions may, in principle, provide additional electronic transitions to enhance energy absorption, their concentration is likely insufficient to compensate for the reduced pyrrole content and increased light scattering from aggregated structures. Thus, a clear trade-off emerges: while TA–Cu modification enhances oxidative activity (ROS generation), it compromises photothermal efficiency.

Besides, these findings illustrate a functional trade-off between oxidative activity and photothermal efficiency (ROS ↑ vs. heat ↓). The TA–Cu coating enhances ROS generation through Fenton-like processes (Cu^2+^ + H_2_O_2_ → •OH + Cu^+^; Cu^+^ + H_2_O_2_ → •OH + OH^−^), but simultaneously reduces the effective PPy content responsible for light-to-heat conversion. This reciprocal relationship underscores a fundamental design challenge for dual-mode PDT/PTT platforms: how to maximize ROS amplification without overly compromising photothermal performance. From a design perspective, this trade-off could be rationally tuned by adjusting coating thickness, controlling TA-to-Cu ratios, or integrating additional plasmonic co-factors (e.g., Au, CuS) to restore or even synergize photothermal efficiency. Moreover, the hollow architecture of PPy@PyCOOH provides favorable internal pathways for both ROS diffusion and light absorption, while the roughened surface introduced by TA–Cu complexation may increase catalytic active sites. Such structural–functional coupling suggests that careful optimization of morphology and surface chemistry can strategically balance oxidative and thermal modalities, thereby enabling more effective and controllable PDT/PTT therapies.

## 4. Critical Limitations and Future Research Directions

### 4.1. Research Limitations

While the development of PPy@PyCOOH and its subsequent surface modification with TA–Cu coatings presents a promising nanoplatform for synergistic photothermal and photodynamic cancer therapy, several limitations should be critically addressed before advancing toward translational applications. These limitations are not only conceptual but also experimental, underscoring areas where further refinement and validation are necessary.

Absence of In Vivo Validation: Although the present study systematically evaluated degradation, ROS generation, and photothermal properties in vitro, the lack of in vivo validation represents a major limitation. Biological systems are inherently more complex, involving factors such as biodistribution, clearance kinetics, immune system interactions, and tumor accumulation efficiency that cannot be fully reproduced in cell-free or in vitro assays. For example, without animal models (e.g., murine xenografts or orthotopic tumor models), it remains unknown whether the observed degradability and ROS-enhancing effects will translate into measurable therapeutic outcomes such as tumor growth inhibition, survival rate extension, or hematological safety parameters (e.g., liver enzyme levels, blood cell counts) [10]. Future work should prioritize such animal-level studies to bridge the gap between in vitro promise and in vivo relevance.

Lack of Active Targeting Capability: The current design does not incorporate any tumor-specific targeting ligands. While the introduction of carboxyl groups in PPy@PyCOOH offers reactive sites for further conjugation, no active targeting strategy (e.g., peptide-based targeting, antibody functionalization, or aptamer binding) was implemented in this study. As a result, the nanoparticles rely solely on passive accumulation via the enhanced permeability and retention (EPR) effect, which is often inefficient and heterogeneous across tumor types. This limitation could lead to nonspecific distribution, reduced therapeutic efficacy, and increased risk of systemic side effects. Integrating active targeting moieties would significantly enhance tumor selectivity and improve the therapeutic index.

Trade-Off Between Photothermal and Photodynamic Functionality: A noticeable limitation of the TA–Cu modification lies in the functional trade-off it introduces. While the coating significantly boosts ROS generation through Fenton-like reactions, it concurrently reduces photothermal conversion efficiency by diluting the PPy core and enhancing interparticle scattering. This reciprocal relationship (ROS ↑ vs. heat ↓) poses a design challenge for dual-mode PDT/PTT systems, where synchronized oxidative and thermal effects are desirable. A mechanistic understanding of how coating thickness, TA-to-Cu ratio, or particle aggregation influences this balance is still lacking. Without such optimization, the dual-mode system may not achieve maximal synergy, and therapeutic outcomes could be compromised [18].

Stability and Storage Concerns: The long-term physicochemical stability of the synthesized nanoparticles was not systematically investigated. Metal–polyphenol coordination bonds are inherently dynamic and prone to hydrolysis or oxidative degradation. As such, prolonged storage could affect structural integrity, dispersibility, and functional reproducibility. Detailed studies on shelf-life, redispersibility after drying or lyophilization, and the effect of storage conditions (e.g., pH, ionic strength, temperature) will be essential to ensure reproducibility in preclinical and eventual clinical applications.

Biosafety and Toxicity Profile: Although PPy-based systems have generally demonstrated good biocompatibility in prior studies, the incorporation of Cu^2+^ ions and polyphenolic coatings introduces potential new safety concerns. These may include altered cytotoxicity profiles, immunogenicity, or off-target ROS-mediated oxidative damage. Importantly, this study did not include standard cytotoxicity assays (e.g., MTT, CCK-8, live/dead staining) or systemic biosafety evaluations (e.g., pro-inflammatory cytokine release, immunogenicity markers) [10]. Without such data, the risk–benefit profile of the nanoplatform remains incomplete.

### 4.2. Future Research Directions

Building upon the foundational work presented here, several promising directions emerge that can guide future research efforts. First and foremost, in vivo studies are essential to validate the therapeutic efficacy, biodistribution, pharmacokinetics, and systemic biosafety of these nanomaterials. Such studies would offer insight into how the nanoparticles interact with complex biological environments and provide a more realistic basis for evaluating their potential clinical utility. In addition to validating performance in animal models, enhancing tumor specificity through surface functionalization represents a critical next step. The carboxyl groups present on PPy@PyCOOH offer a convenient chemical handle for the conjugation of targeting ligands. By attaching tumor-homing molecules such as folic acid, RGD peptides, or aptamers, the nanoparticles could achieve receptor-mediated uptake, improving their therapeutic index and reducing unintended accumulation in healthy tissues.

The optimization of the TA–metal coordination layer also warrants further investigation. While the current use of Cu^2+^ significantly boosts ROS production, it diminishes photothermal conversion. Fine-tuning the balance between metal content, coordination density, and polymer integrity—potentially through the incorporation of alternative metal ions like Fe^3+^ or Mn^2+^—could help overcome this trade-off. Such adjustments may allow researchers to tailor the dual functionality of the platform for different therapeutic scenarios. Another important area for exploration involves understanding the biodegradation kinetics and clearance pathways of these nanoparticles in vivo [20]. Detailed analysis of enzymatic breakdown, organ-specific accumulation, and elimination through renal or hepatic routes would shed light on the materials’ long-term behavior and potential biosafety concerns. This is particularly crucial when considering repeated or long-term therapeutic regimens.

Expanding the platform’s responsiveness to other tumor-relevant stimuli offers additional flexibility. Incorporating pH-, redox-, enzyme-, or temperature-sensitive linkers could enable more precise spatiotemporal control over drug release and therapeutic activation. Such multi-stimuli responsive systems would be better suited for navigating the heterogeneous and often harsh conditions of the tumor microenvironment. Finally, while this study focused on cancer therapy, the multifunctional properties of the synthesized nanoparticles open opportunities for broader biomedical applications. Their strong photothermal response and ROS-generating capacity could be valuable in antimicrobial treatments, biofilm disruption on medical devices, wound healing, or even non-oncologic inflammatory diseases. Investigating these potential cross-disciplinary applications would further demonstrate the versatility and utility of the platform.

## 5. Conclusions

PPy NPs have emerged as a promising new generation of photothermal agents and have attracted considerable attention in recent years. They have been widely applied in biomedical imaging and cancer theranostics. In this study, PPy nanospheres were synthesized via oxidative polymerization using FeCl_3_·6H_2_O as the oxidant. Subsequently, a template-free method was employed in which pyrrole and pyrrole-3-carboxylic acid (Py-3-COOH) were copolymerized using a FeCl_2_/H_2_O_2_ oxidation system, resulting in the formation of hollow nanospheres. The in vitro degradation behavior of PPy@PyCOOH and pristine PPy was comparatively evaluated under varying concentrations of hydrogen peroxide. The results revealed that the hollow PPy@PyCOOH nanospheres exhibited a significantly faster degradation rate than solid PPy. Therefore, PPy@PyCOOH was selected for further surface modification in subsequent experiments.

To enhance its functionality as a nanobiomaterial, tannic acid (TA) was introduced to the PPy@PyCOOH surface, forming a metal–polyphenol coordination network through chelation with copper ions. Subsequent experimental results demonstrated that the TA-coated nanoparticles exhibited markedly enhanced ROS generation capability under identical conditions compared to uncoated PPy@PyCOOH. However, the photothermal heating efficiency of the TA-coated nanoparticles was lower than that of the uncoated PPy@PyCOOH, likely due to compositional dilution or light absorption interference. Despite this, both types of nanoparticles showed excellent photothermal stability, with negligible variation in heating rate and maximum temperature over multiple heating–cooling cycles.

## Data Availability

The original contributions presented in this study are included in the article. Further inquiries can be directed to the corresponding author.
